# Texture Synthesis Based Thyroid Nodule Detection From Medical Ultrasound Images: Interpreting and Suppressing the Adversarial Effect of In-place Manual Annotation

**DOI:** 10.3389/fbioe.2020.00599

**Published:** 2020-06-17

**Authors:** Siqiong Yao, Junchi Yan, Mingyu Wu, Xue Yang, Weituo Zhang, Hui Lu, Biyun Qian

**Affiliations:** ^1^School of Life Sciences and Biotechnology, Shanghai Jiao Tong University, Shanghai, China; ^2^School of Electronic Information and Electrical Engineering, Shanghai Jiao Tong University, Shanghai, China; ^3^Hongqiao International Institute of Medicine, Shanghai Tong Ren Hospital and Clinical Research Institute, Shanghai Jiao Tong University School of Medicine, Shanghai, China

**Keywords:** ultrasound medical image, nodule detection, image inpainting, deep learning, variational information bottleneck

## Abstract

Deep learning method have been offering promising solutions for medical image processing, but failing to understand what features in the input image are captured and whether certain artifacts are mistakenly included in the model, thus create crucial problems in generalizability of the model. We targeted a common issue of this kind caused by manual annotations appeared in medical image. These annotations are usually made by the doctors at the spot of medical interest and have adversarial effect on many computer vision AI tasks. We developed an inpainting algorithm to remove the annotations and recover the original images. Besides we applied variational information bottleneck method in order to filter out the unwanted features and enhance the robustness of the model. Our impaiting algorithm is extensively tested in object detection in thyroid ultrasound image data. The mAP (mean average precision, with IoU = 0.3) is 27% without the annotation removal. The mAP is 83% if manually removed the annotations using Photoshop and is enhanced to 90% using our inpainting algorithm. Our work can be utilized in future development and evaluation of artificial intelligence models based on medical images with defects.

## 1. Introduction

Medical imaging is one of the most important sources of clinical diagnosis and clinical practice has accumulated and is currently generating huge amount of high-resolution medical imaging. However, the evaluation and interpretation of medical image highly depend on specialists with qualified skills which is quite limited resource, especially in the developing countries. The blooming of deep learning has open unprecedented opportunity for adopting computational approach toward automatic and accurate processing of medical images. Clinical applications are from image augmentation, segmentation (Badrinarayanan et al., 2017; Poudel et al., 2019), lesion detection to diagnosis, risk stratification and clinical decision support (Cai and Vasconcelos, 2018; Li Z. et al., 2018; Redmon and Farhadi, 2018). Deep learning requires considerable amount of labeled medical image datasets to train a usable model. It's also worth mentioning that ultrasonic inspection (UI) has some particular advantages in practice, by dynamic multi-trials, the area of interest can be effectively identified, leading to better (qualitative) measurement of size, quantity, diolame, calcification, and the relationship between different tissues of organizations. All such information is critical and helpful to the early screening and guidance before and after surgery and other medical treatment. The application of deep learning in ultrasound images is expected to be used in some areas like identification of benign and malignant nodules or the nodules detection.

For the ultrasound images, we have identified an important while ignored problem that in current clinical practice of ultrasound images, such as the detection images of thyroid nodule, the clinician will mark the position of the nodule with marker and measure the size of the nodule in the meantime (usually called calibration marker). Because of the limits and routines of mainstream device and clinical setting, the markers are often inseparable from the raw image. In another word, the raw image has been permanently corrupted which is irrecoverable. This is no surprise as before the wide deployment of automatic detection for ultrasound images, the images are mostly scrutinized by doctors. Moreover, we find this limitation is practically ubiquitous in many other areas such as breast.

While in the real application scenario—utilizing this model to automatically detect thyroid nodule and reducing the clinician's workload—these markers would not exist. As a result, when prediction models trained using such annotated images can be misled by the markers leading to severe overfitting on true diagnose setting where the data for detection is prospective. The prediction accuracy was quite low when training with these images with markers. To the best of our knowledge, little study has been conducted on this issue, which in fact has been a direct block for applying off-the-shelf detection models to the ultrasound images.

Until now, clinical practice has accumulated huge amount of high-resolution medical imaging which can be used for clinical research. These medical image data should have tremendous value to be utilized in training machine learning models which are presumed to be similar to those in future applications. However, because of the problem about the manual annotations in medical images made by clinicians, these data can not be directly used in training model. They can only be used for statistical study of existing results. Therefore if we want to use a lot of historical data to train the model, in order to achieve the effect of generalization in the new data, especially the detection task, which has a strong offset to the image feature selection, we have to focus on the processing of historical data. To thoroughly present the concept and empirically test our method, we take the nodule detect task in thyroid ultrasound image as an example.

By taking a domain adaption perspective, we try to fill the gap between vanilla images and those with manual annotations. In Zhang et al. (2017), author suggest two general approaches for visual domain adaptation: data-centric and subspace-centric method. On one hand, we developed a data centric method using inpainting algorithm which automatically remove the manual annotations and recover the original images. On the other hand, we also explored some model modifications to filtering out the features related to the markers.

In a nutshell, the main highlights of this paper are as follows:

- We find the important while often less studied problem: most existing real-world ultrasound imaging and annotation systems are designated by default to keep the intrusive annotation layered on the raw image which causes non-negligible the overfitting problem which is further verified by our empirical study.
- We address the problem of computational and more specifically deep learning based thyroid nodule detection. Note our approach involves multiple techniques in machine learning and computer vision, with a focus on solving real-world problems.
- In particular, we devise a few of data preprocessing techniques for mitigating the disturbance of markers, which to our knowledge have been little done on ultrasound medical images (especially for the thyroid ones). In particular, experimental results on the collected real-world images show that the method of using impainting can get the best effect. Also the performance of network feature optimization is further enhanced by the variational information bottleneck approach, which aims to extract compact and robust features related to the supervised task (nodule classification in this paper).

The rest of this paper is organized as follows: section 2 reviews the related work on thyroid nodule analysis for medical images and general methods for image inpainting. Section 3 describes the technical details of our approach, including the detection Pipeline, inpainting based preprocessing, random mixup of markers for preprocessing and deep variational information bottleneck for classification. The details of data collection and data description, image inpainting and model modification are shown in section 4. Section 5 concludes discussion, concluding remarks of this paper and future work.

## 2. Related Work

In this section, we review the related work from two perspectives: (i) Thyroid nodule analysis for medical images; and (ii) General methods for image inpainting. These areas are technically related to our work.

### 2.1. Thyroid Nodule Analysis for Medical Images

The authors in Li et al. (2019) have shown the adoption of the off-the-shelf deep network models i.e., ResNet-50 (He et al., 2016) and DarkNet-10 (Redmon, 2013-2016) can achieve promising accuracy (above 90%) in terms of benign and malignant classification for thyroid nodule. While classification is often regarded less difficult than detection which is the focus of this paper. In Li H. et al. (2018), the authors have adopted and further improved the Faster-RCNN detector (Ren et al., 2015) for the task of mastoid tumor detection on thyroid. An automatic and scalable pipeline for analyzing ultrasonic cardiogram has been established in Zhang et al. (2018), and the components include area of interest detection, segmentation, quantitative study on the structure and function, and disease diagnose etc. We also mention the so-called DeepLesion dataset has been introduced in Yan K. et al. (2018) whereby deep network is used to extract the deep features for different lesions.

We use the thyroid images for case study, and go beyond the direct application of the existing object by considering the intrusive markers.

### 2.2. General Methods for Image Inpainting

Image inpainting has been a fundamental tool for image or video editing, rendering and other image processing settings (Barnes et al., 2009; Newson et al., 2014). The early learning-free works (Levin et al., 2004; Barnes et al., 2009) borrow the idea from texture synthesis (Efros and Leung, 1999; Efros and Freeman, 2001) to address the image inpainting task. The basic assumption is that the background texture can be used to fill up the defected area. In another word, they have difficulty in generating complex and arbitrary image patches. Recently leaning based generative models e.g., generative adversarial networks (Goodfellow et al., 2014) have gain increasing popularity for their expressiveness, and have also been applied in image inpainting (Li et al., 2017). Further improvement is made in a very recent work (Yu J. et al., 2018) whereby the boundary artifacts are effectively addressed by introducing a new context-aware layer, which can compensate the discontinuity between the surrounding content and the inpainting part. Also, Wasserstein GAN (Arjovsky et al., 2017) has been used to stabilize the training and avoids mode collapse.

In this work, image inpainting is used as a building block for preprocessing on the training dataset to dismiss the effect of over-layered markers. Our work differ from previous studies in that the objective of our model is not merely restore the original image but also minimize the effect on the other computer vision task like object detection.

## 3. Approach

**Figure 1:**
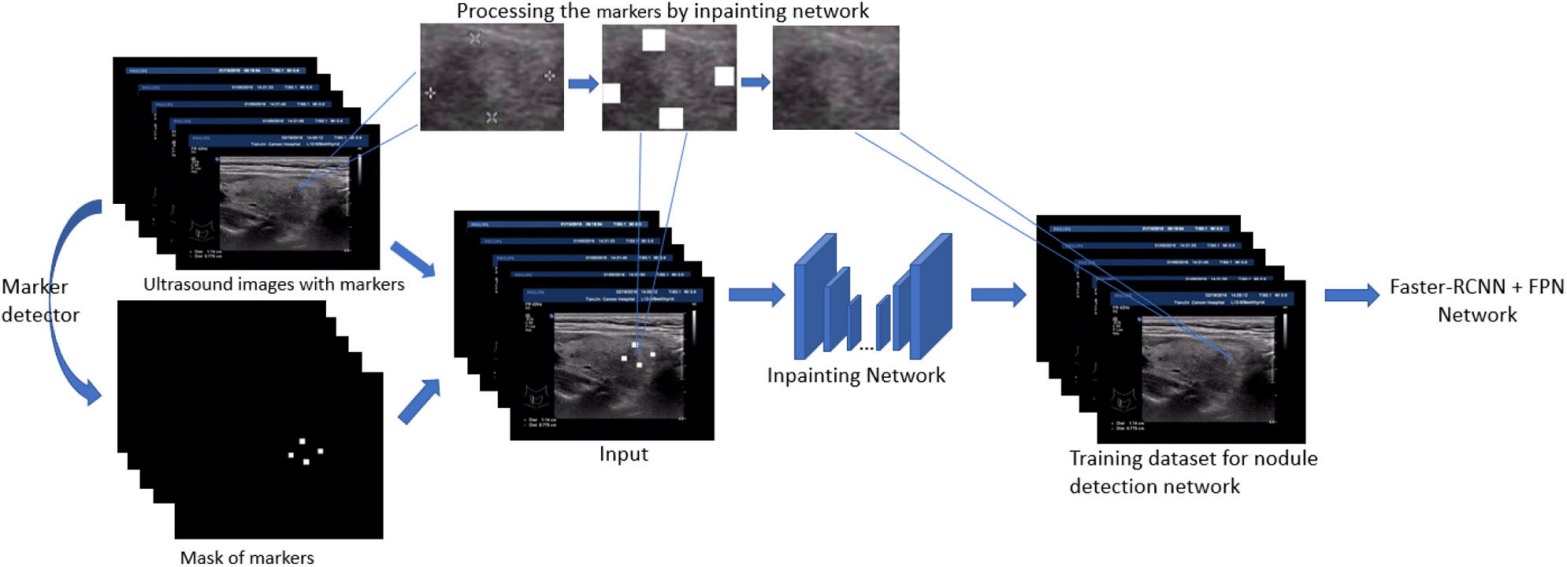
Pipeline of nodule detection for training set with image inpainting preprocessing.

In this section, we present the main techniques used in the paper. First, the detection models based on Feature Pyramid Networks (FPN) (Lin et al., 2017) and faster-RCNN (Ren et al., 2015) are our backbone models and fulfill the original task: thyroid nodule detection. Second, to filling the gap between the training image and test image in nodule detection, a generative network based inpainting method (Yu J. et al., 2018) is employed to remove and replace the markers with a natural texture which minimize the effect on object detection. Based on this off-the-shelf method, we further generalize our strategy by either removing or adding markers. The overview of our inpainting based preprocessing for training detection model is shown in Figure 1. Finally, among several model modifications, the utility of VIB (variational information bottleneck) is explored and discussed.

### 3.1. Detection Pipeline

**Figure 2:**
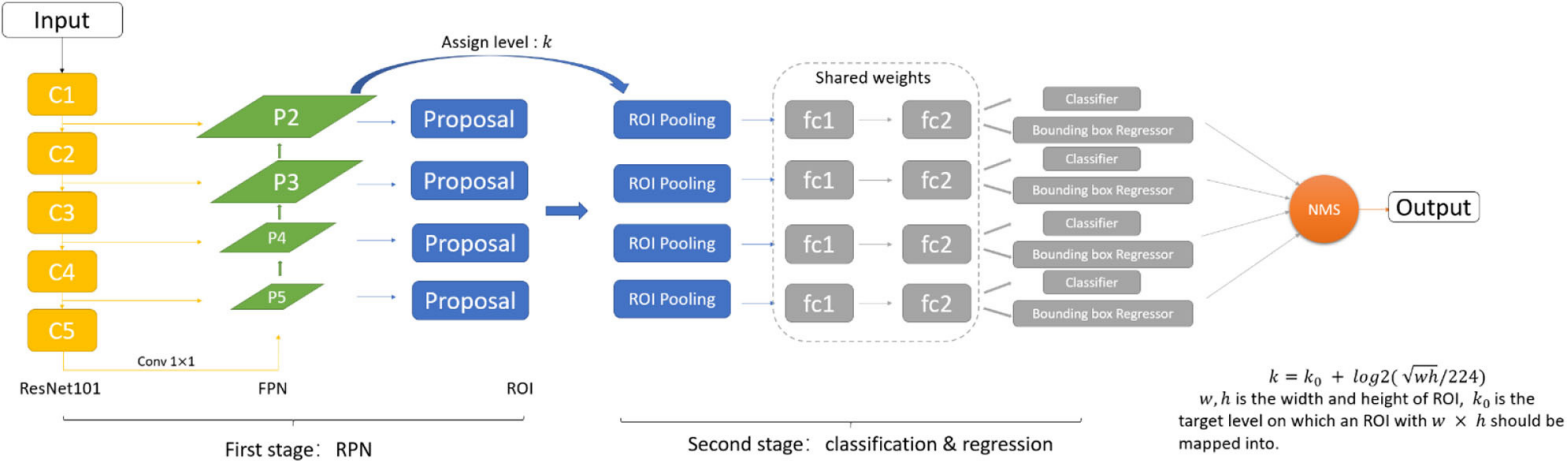
The FPN based Faster-RCNN network structure.

As shown in Figure 2, we adopt the FPN based Faster-RCNN network for nodule detection. Specifically, Faster-RCNN is a two-stage detection network. In the first stage, it generates the candidate bounding box through a Region Proposal Network. We use the ResNet101 and Feature Pyramid Network as the feature extractor to generate the feature map. FPN is an efficient method to extract features of each dimension in the images. It can significantly improve the performance of detection network in the multi-scale nodule detection problem. By using FPN structure, we get 4 scales of feature through the backbone, and further, 4 ROI pooling will give the feature maps corresponding to candidate regions generated from the RPN. In the second stage, we can get the final detection results through the classification and regression network.

### 3.2. Inpainting Based Preprocessing

In this paper, we use the method of inpainting to erase the annotated intrusive markers layered around the nodule area and to synthesize visually realistic and semantically reasonable pixels for the missing regions, which coexist with the existing ones. Compared with the traditional filling method of missing pixels of an image, the method we use (Yu J. et al., 2018) has made the following two characters.

- Global and local Wasserstein Generative Networks (WGANs) (Arjovsky et al., 2017) are introduced to overcome the problems of the disappearance of training gradient descent and the collapse mode in traditional method of GAN.
- Context attention model is introduced. The convolution neural network processes the image features with local convolution kernel layer by layer, so it has no effect on getting the features from the far space information. In order to overcome this limitation, attention mechanism and a new contextual attention layer is introduced in the deep generation network.

**Figure 3:**
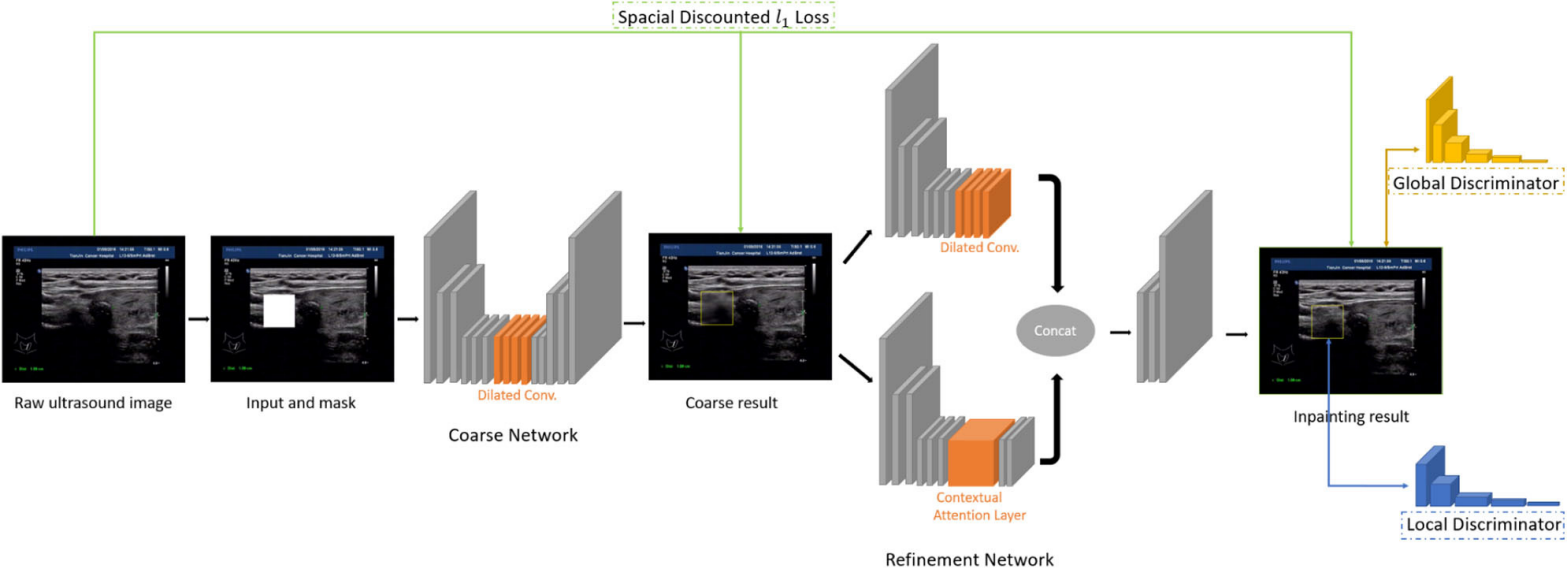
The inpainting network structure.

As illustrated in Figure 3, the network consists of two main parts: generating network to restore the images (RGB images with binary mask indicating area of interest) and two classification networks (global discriminator and local discriminator) to identify whether the generated image is consistent with the original image. The difference is that generation network we use is divided into two steps. In the first step, the Coarse Network generates a rough result from the input ultrasound image, and only the reconstruction loss is employed during the training process. Then in the second step, the Refinement Network will be trained with reconstruction loss, together with the global and local WGAN-GP adversarial loss. As a result, details will be restored during this process. In addition, dilated convolution is employed in both coarse and refinement network to increase the receptive field of convolution kernel while keeping the number of parameters and size of output feature map unchanged.

### 3.3. Random Mixup of Markers for Preprocessing

In view of the pixel information and relative position information that the markers contain, we weaken the influence of the markers in two ways:

The first method is to add regular markers in the ultrasound images. We randomly add pseudo areas of nodule outside the region of true nodule. The pseudo areas pf nodules should contain all the features of the ground truth except the feature of nodule texture. Therefore, we should imitate how doctors label suspicious regions. The markers are mostly located at the point of tangency between the four sides of the largest external rectangle of the nodule and the nodular contour, which are usually in groups of two or four. The process of adding a pseudo area of nodule is as follows:

- Determine the size of the nodule and ensure that its length and width do not exceed that of the largest nodule in the samples.
- Determine the proper location of pseudo areas of nodule and ensure the IoU value of its region and every existing nodules is 0.
- Determine the location of markers. In one case, there is a marker on the left and right sides of the rectangle. In the other case, there is a marker on the each side of the rectangle. Each marker located at a random position at one side.

The second method is to destroy the structure of markers' position and then add markers at random position in the ultrasound images. We keep only the parts of each set of markers. For the annotation that contains 2 markers, we keep one marker and add markers at random locations outside of the nodule region. For the annotation that contains 4 markers, we randomly keep 1 to 3 markers and add markers at random locations outside of the nodule region.

### 3.4. Deep Variational Information Bottleneck for Classification

One important observation in this paper is that the detected region proposals for candidate nodule obey different distribution between the training dataset and the testing dataset. This can incur instability and poor performance on classifying the nodule and non-nodule areas. More specifically, for practical reason, the historically collected ultrasound images often are recorded with in-place over-layered markers on the fly, which are annotated by doctors. In contrast, the ultrasound images for detection are first-hand, without any annotation for automatic detection. As a result, the training data has the data leak issue which is contaminated and there exhibits a strong bias for easy overfitting because the markers are strongly related to nodule areas in space. In another word, there is a domain shift between the training and testing set.

To mitigate this issue, in this paper we adopt the deep variational information bottleneck (Alemi et al., 2017) technique. Based on the information bottleneck theory (Saxe et al., 2019), the classification network equipped with this technique can automatically ignore the irrelevant information with classification by extracting a more compact feature representation via the supervised information.

By the neural network with information bottleneck, the input *X* is encoded as *Z*. The focus is to maximize the mutual information between *Z* and label *Y*, so that classification can be performed using the encoding information *Z*:

(1) $$\begin{array}{l} I\left(Z,Y\right)=\int \text{d}z\text{d}yp\left(y,z\right)\log\frac{p\left(y|z\right)}{p\left(y\right)} \end{array}$$

where *X, Y, Z* are random variables and *x, y, z* are instances of random variables.

According to information bottleneck theory, the ultimate optimization task is to maximize the value of *R*_*IB*_ (Strouse and Schwab, 2017):

(2) $$\begin{array}{l} R_{IB}=I\left(Z,Y\right)-\beta I\left(Z,X\right) \end{array}$$

where β is a Lagrange multiplier. It means the mutual information between *X* and *Z* should be limited in a information constraint to ensure a lower feature complexity and the mutual information between *Z* and *Y* should be as large as possible to ensure *Z* can represent *X* adequately.

In order to add this monitoring mechanism to classification in a tractable and differentiable way, we adopt the solution proposed by Alemi et al. (2017).

Since *p*(*y*|*z*) in (1) is difficult to compute, the variational approximation *q*(*y*|*z*) is used to replace it. The Kullback Leibler divergence of *p*(*y*|*z*) and *q*(*y*|*z*) is positive, it can be given as:

(3) $$\begin{array}{l} I\left(Z,Y\right)\geq \int \text{d}x\text{d}y\text{d}zp\left(x\right)p\left(y|x\right)p\left(z|x\right)\log q\left(y|z\right) \end{array}$$

where $p\left(z|x\right)=N\left(z|f_{e}^{\mu }\left(x\right),f_{e}^{\Sigma }\left(x\right)\right)$ is assumed to the encoder. *f*_*e*_ is ResNet50 without the last fully connection layer in this paper. It outputs 2K dimension vector, where the first K dimension is used to encode μ and the last K dimension is used to encode σ after a softplus layer.

(4) $$\begin{array}{l} I\left(Z,X\right)=\int \text{d}z\text{d}xp\left(x,z\right)\log\frac{p\left(z|x\right)}{p\left(z\right)} \end{array}$$

Similar to the steps above, the variational approximation of *p*(*z*), *r*(*z*) is introduced to replace it. Then *R*_*I*_*B* has the lower bound:

(5) $$\begin{array}{l} R_{IB}\geq \int \text{d}z\text{d}yp\left(y,z\right)\log\frac{p\left(y|z\right)}{p\left(y\right)}-\beta \int \text{d}x\text{d}zp\left(x\right)p\left(z|x\right)\log\frac{p\left(z|x\right)}{r\left(z\right)} \end{array}$$

where $q\left(y|z\right)=S\left(y|f_{d}\left(z\right)\right)$ is assumed to the decoder.S() is the softmax function, *f*_*d*_ is a mapping from z to the logits of 2 classes in our experiment and r(z)=N(z|0,I). Using the empirical estimation model of *p*(*x, y*), Alemi et al. (2017) got the formulation as a loss function in classification task.

## 4. Results

### 4.1. Data Collection and Data Description

**Figure 4:**
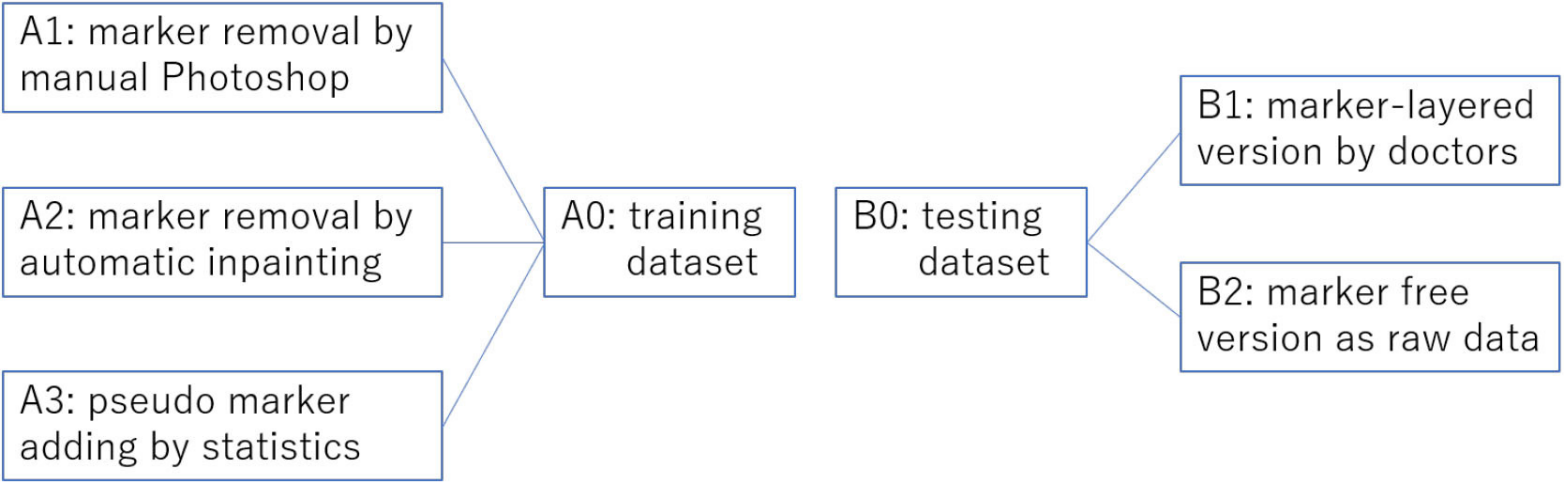
Overview of the training set preprocessing practices and the corresponding two versions of testing sets without preprocessing. This overview corresponds to Table 1.

We collected overall 2,459 thyroid ultrasound images of 300 patients from a tertiary hospital in China. Among these ultrasound images, as shown in Figure 4, 1,791 (set A0) are historical data with clinician's manual annotation on it and served as the training data for the object detection model. The 3 image data sets generated from A0 using three different processing method are A1, A2, A3, respectively. The other 668 are purposely collected marker free testing set B2 which is the same situation as the thyroid nodule detection module worked on in real world clinical setting. B1 consists of images from B2 with later annotated markers by clinicians. Therefore, there are 1,336 images as testing data(set B0). B2 mimics an improper test set often used by medical AI researchers which differs significantly from the realistic one (B2). Ethics are approved by the IRB of the local hospital.

In the second setting for variational bottleneck method evaluation, in order to optimize the feature map of the network, note only classification is involved based on given image crops. Specifically, the training set consists of 872 image crops without nodule (i.e., negative samples), in addition with 895 crops with real nodule on each of them (i.e., positive samples). The testing set contains 865 negative crops together with 896 positive crops for classification. We keep the training set as is while design three cases for the testing set: (i) keep the 896 positive crops in the test set with raw manual markers as is, and randomly add pseudo markers to each of the 865 negative crops; (ii) keep the 896 positive crops in the test set with raw manual markers as is, and also keep the 865 negative crops as is without mark; (iii) use the marker-free version for the 896 positive samples and keep the 865 negative crops as is. These cases are shown in Table 2 for the description of the testing set.

### 4.2. Image Inpainting

We study three ways to preprocess the raw ultrasound images to verify the effect of the intrusive markers: (i) remove the markers by Photoshop manually; (ii) remove the markers by an automatic computational inpainting approach (Yu J. et al., 2018); (iii) randomly add pseudo markers to, and remove the real markers from the marker-layered image. These three protocols are reflected in Table 1.

**Table 1:** Nodule detection performance by mAP, recall, and F-score under two settings for IoU, by using different settings in terms of the way of preprocessing on training set, and the testing set version.

**Dataset**		**IoU = 0.4**			**IoU = 0.3**		
**Training preprocess**	**Testing version**	**mAP**	**recall**	**F-scr**	**mAP**	**Recall**	**F-scr**
A0:marker-layered	B1:marker-layered	98.00	98.20	98.12	99.49	99.55	99.47
A0::marker-layered	B2:marker-free	24.10	41.44	31.71	27.49	46.10	35.27
A1:removed by PS	B1:marker-layered	65.21	60.06	70.55	73.81	70.42	75.71
A1:removed by PS	B2:marker-free	69.22	61.71	71.98	83.37	68.92	81.53
A2:removed by IP	B1:marker-layered	80.73	81.68	82.61	89.00	90.09	89.42
A2:removed by IP	B2:marker-free	84.31	80.48	83.23	89.61	83.48	86.33
A3:randomly	B1:marker-layered	95.54	95.80	95.58	98.64	98.20	97.98
Added&removed	B2:marker-free	72.60	65.17	74.89	76.16	68.32	77.05

*Note that the training set is always a marker-layered(A0) version while we have collected two versions for the testing set i.e., B1 and B2. Testing on the B2 version is more realistic in practice. Note the training set A3 is fixed to a collection of 872 negative crops with added pseudo markers in addition with 895 positive crops layered with raw markers. PS denotes Photoshop tool, and IP denotes image inpainting technique. See Figure 4 for the corresponding setting for training set*.

We adopt the widely accepted metrics for evaluating the performance of our detection approach, namely recall, precision, mean average precision (mAP) and F1-score. Given the number of true positives (TP), true negatives (TN), false positives (FP) and false negatives (FN), the precision is obtained by:

(6) $$\begin{array}{l} P=\frac{TP}{TP+FP} \end{array}$$

The recall is given as follows:

(7) $$\begin{array}{l} R=\frac{TP}{TP+FN} \end{array}$$

The F-score, as a summary statistic of precision and recall, is obtained as follows:

(8) $$\begin{array}{l} F=\frac{2\times P\times R}{P+R} \end{array}$$

Suppose P(*R*_0_) is the maximum value of *P* when *R* ≥ *R*_0_, then the average accuracy for current class *i* is defined by:

(9) $$\begin{array}{l} AP_{i}=\int_{0}^{1}P\left(R_{0}\right)dR_{0} \end{array}$$

For *m* classes, the mean average precision is given as follows:

(10) $$\begin{array}{l} mAP=\frac{1}{m}\overset{m}{\underset{i=1}{\sum }}AP_{i} \end{array}$$

**Figure 5:**
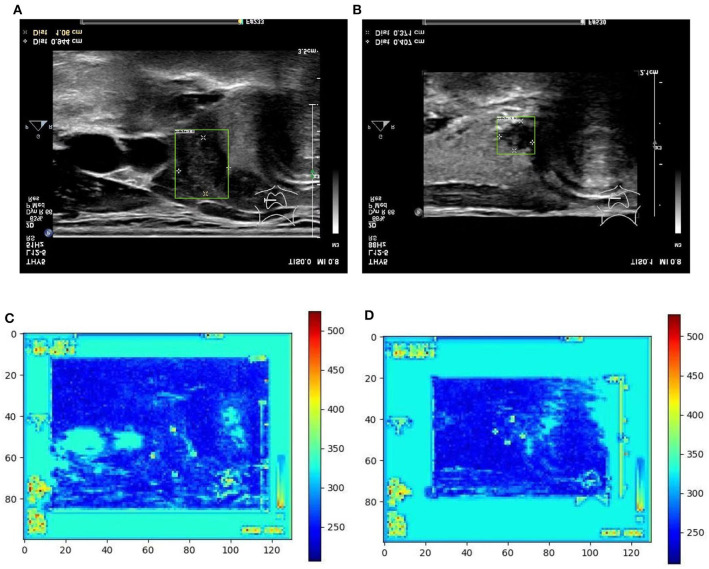
Two examples (from left to right, correspond to the first two-rows in the content of Table 1) of nodule areas which are marker-layered as detected by the bounding box in the ultrasound thyroid image, and their corresponding response heat map as visualized by the feature map in the detection part of the networks. Note the heat maps are computed on the model trained by samples whose markers are kept. Best viewed in color. **(A)** Nodule in ultrasound thyroid image. **(B)** Nodule in ultrasound thyroid image. **(C)** Corresponding response heat map. **(D)** Corresponding response heat map.

**Figure 6:**
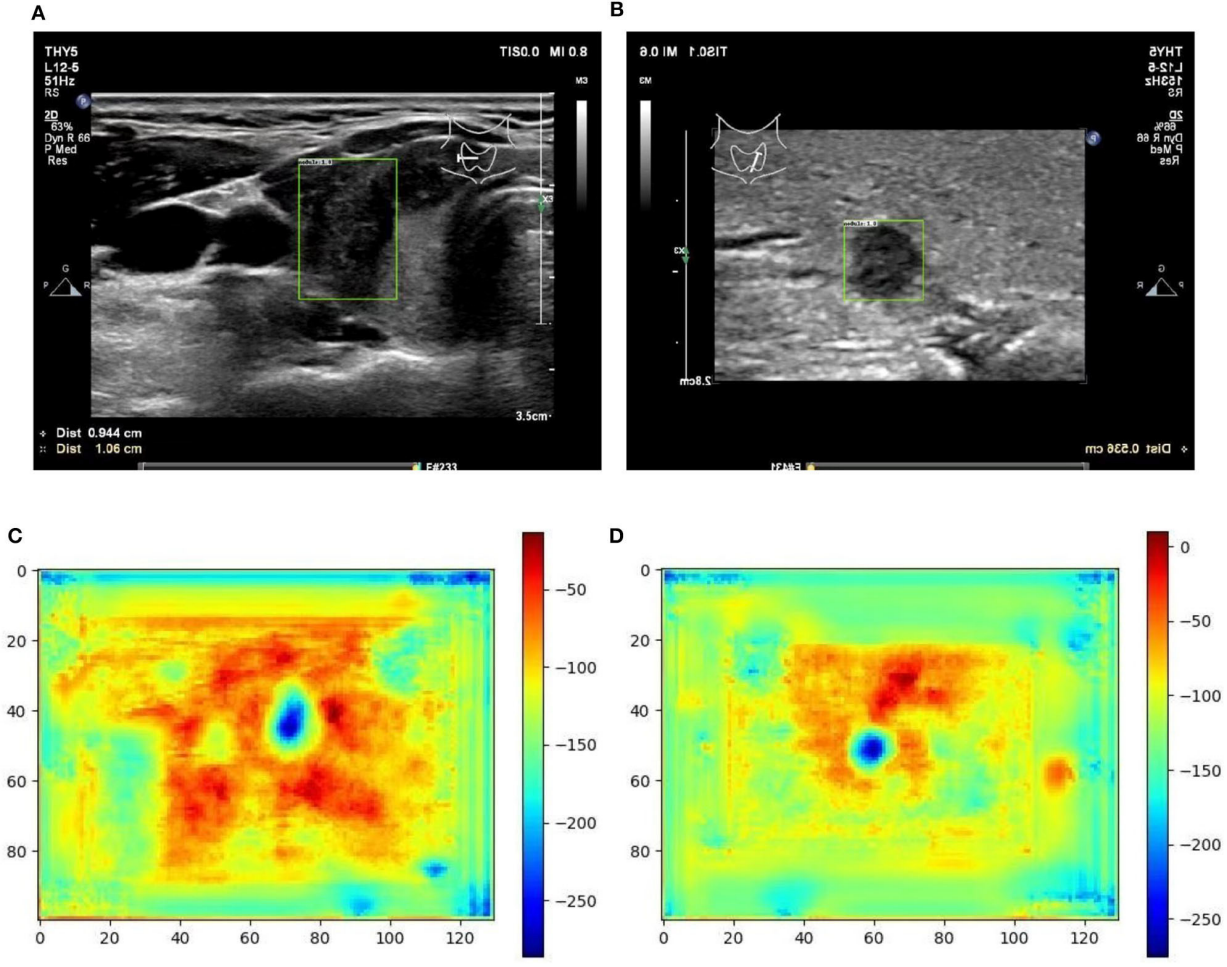
Two examples (from left to right, correspond to the third two-rows in the content of Table 1) of nodule areas which are marker-free as detected by the bounding box in the ultrasound thyroid image, and their corresponding response heat map as visualized by the feature map in the detection part of the networks. Note the heat maps are computed on the model trained by samples whose markers are removed by manual (IP). Best viewed in color. **(A)** Nodule in ultrasound thyroid image. **(B)** Nodule in ultrasound thyroid image. **(C)** Corresponding response heat map. **(D)** Corresponding response heat map.

From Table 1 it can be found that given the marker-layered training samples, the detection performance degenerates significantly when we switch the testing set from marker-layered samples to marker-free ones. This phenomenon is also illustrated in Figure 5 that the markers are highlighted in the detection feature map response (heat map) which can mislead the detector in real-world applications when the image samples are marker-free. We analyze this is caused by overfitting the marker pattern associated with the real nodules in the training set. Then we try to remove the markers from the training set either by manual (Photoshop tool) or by an automatic inpainting model (Yu J. et al., 2018), we find the degeneration becomes less notable while the performance can be worse than the overfitted version [slightly for the inpainting (IP) based method]. This phenomenon is also illustrated in Figure 6. Finally we show that adding and removing markers to the marker-layered training set samples leads to performance worse than the marker-removal strategies as shown in the bottom two rows in Table 1.

In particular, we show that the inpainting based approach performs much better than the manual PS-based methods, under the same settings of the other part in the algorithm pipeline. This indicates the usefulness for using inpainting for marker-layered training and marker-free detection. More importantly it is free from manual labeling and fully automatic for training. In our analysis, we conjecture the promising performance of inpainting based method can be attributed to following reasons: (i) our adopted learning based inpainting model (Yu J. et al., 2018) can more effectively deal with the challenging and blur ultrasound medical images by adaptively extracting the relevant features to the marker-layered area, even they are from other regions away from the markers or from different image samples (in training set); (ii) the inpainting model consists of a series of state-of-the-art building blocks, including the inpainting network enhancement, global and local WGAN and spatial reconstruction loss, to stabilize and speedup the training of the inpainting model and finally the competitive inpainting results.

**Figure 7:**
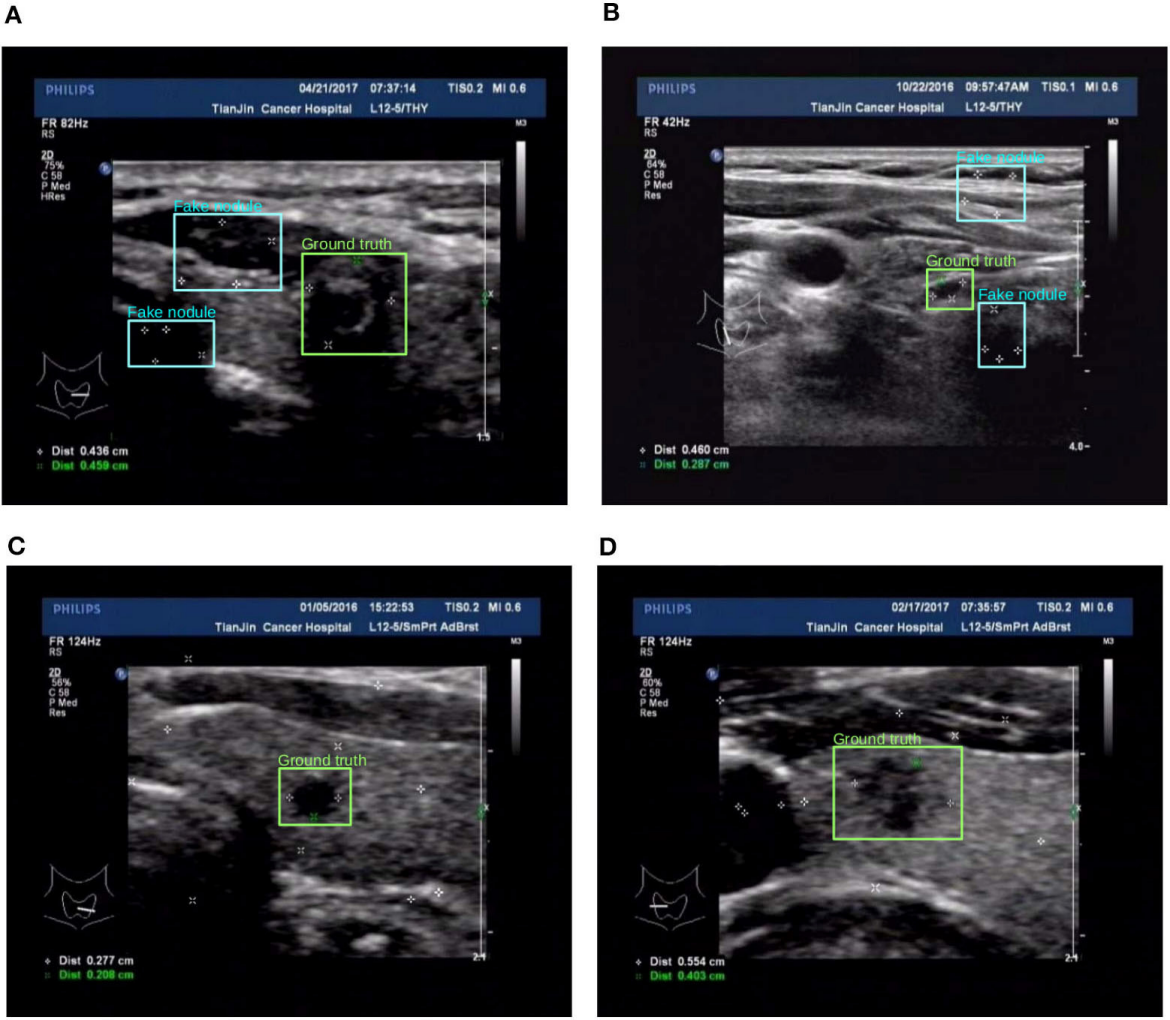
Two examples (from left to right, corresponding to the forth two-rows in the content of Table 1) for the comparison of two different training set preprocessing techniques i.e., randomly adding pseudo markers vs. randomly adding and remove pseudo markers. Best viewed in color. **(A)** Poor detection results. **(B)** Poor detection results. **(C)** Effective detection results. **(D)** Effective detection results.

The third approach is reflected in the last two rows in Table 1, the details are as follows: for the preprocess “randomly added,” it is implemented by adding a few of marker groups in random position outside the nodule area. The marker group is randomly generated with 2 or 4 markers, with reasonable random sizes according the regular annotation practice by doctors. For the preprocess “added&removed,” it is implemented by randomly removing 1 to 3 markers by Photoshop, and meanwhile adding a certain number of markers randomly outside the nodule area. For the random mixture part, it is shown that it can to some extent improve the generalization ability from marker-layered samples to marker-free ones. But it is not as effective as the image inpainting based method. The image samples for the comparison of these two different training set are shown in Figure 7.

### 4.3. Model Modification

The evaluation for our variational information bottleneck method for nodule classification is shown in Table 2. Given a fixed training set consisting of 872 negative crops with added pseudo markers, and 895 positive crops layered with raw markers, one can find that the VIB technique can notably stabilize the performance with the vanilla ResNet50 model, no matter how the testing set is manipulated by different means. This ablation study suggests the improved robustness by VIB technique with promising potential.

**Table 2:** Binary nodule classification accuracy using different settings in terms of the models, testing set version and preprocessing on the negative samples of the testing set.

**Model**	**Case**	**Testing set (# of crops)**		**Accuracy (%)**
		**Negative (865) Processing**	**Positive (896) version**	
ResNet50	Case 1	Pseudo marker added	Marker-layered	98.41
	Case 2	No pseudo marker added	Marker-layered	76.67
	Case 3	No pseudo marker added	Marker-free	64.46
ResNet50 +VIB	Case 4	Pseudo marker added	Marker-layered	95.41
	Case 5	No pseudo marker added	Marker-layered	92.96
	Case 6	No pseudo marker added	Marker-free	95.85

*Note the evaluated method is the variational information bottleneck classification model with the ResNet50 as the backbone for cropped image samples as input. No detection is involved in the table*.

## 5. Discussion, Concluding Remarks, and Future Work

In this paper, we have pointed out an important data leak issue for intrusive annotated medical images, such as the ultrasound thyroid nodule images as has been studied in this paper. Specifically the association of the nodule and the manual annotations layered on the raw images by doctor can cause overfitting of the supervised networks, making their performance significantly degenerate on raw ultrasound medical images without markers. This problem is practically important as almost all existing thyroid nodule images are annotated in such an intrusive way while the detection model need to handle marker-free images in practical use.

In this paper, different preprocessing techniques on the raw images with annotated markers have been developed and we address thyroid nodule detection based on these techniques including: (i) manually remove markers by Photoshop; (ii) remove markers by deep network based inpainting model; (iii) mix the raw markers by adding pseudo and removing true markers; and finally (iv) a variational information bottleneck based classification model based on information theory which is known more robust against domain adaption problem. Here the domain differs if the markers are layered or not. We evaluate the performance of these techniques using real-world ultrasound thyroid nodule images via ablation study and comprehensive evaluation. It verifies that the layered marks can cause significant overfitting issue and removing these markers is a direct and effective way of solving this issue. Using the strategy of disturbing the raw markers works but not as well as the complete removal. Finally, we verify the VIB approach is a robust algorithm against the effect of markers and it performs stably together with different preprocessing strategies.

For future work, we aim to continuously collect images with and without intrusive markers to further study our model—which may take years of efforts. We will also explore the opportunity for collecting other kinds of ultrasound medical images such as lung to further verify the universality of our approach. From the technical perspective, we would explore the matching based methods (Yan J. et al., 2018; Yu T. et al., 2018), especially multiple image matching (Yan et al., 2015a,b, 2016) for ultrasound image registration which can help global alignment and nodule localization of multiple images.

## Data Availability Statement

The datasets generated for this study will not be made publicly available for privacy reason.

## Author Contributions

JY, HL, and WZ contributed conception and design of the study. SY, JY, MW, and XY contributed to experimental process and evaluated and interpreted model results. WZ, BQ, and JY obtained funding for the project. WZ and BQ provided clinical guidance. SY, JY, and WZ drafted the manuscript. All authors contributed to manuscript revision, read and approved the submitted version.

## Conflict of Interest

The authors declare that the research was conducted in the absence of any commercial or financial relationships that could be construed as a potential conflict of interest.
